# Role of muscle FOXO gene in exercise against the skeletal muscle and cardiac age-related defects and mortality caused by high-salt intake in *Drosophila*

**DOI:** 10.1186/s12263-023-00725-2

**Published:** 2023-03-30

**Authors:** Deng-tai Wen, Ying-hui Gao, Jingfeng Wang, Shijie Wang, Qi Zhong, Wen-qi Hou

**Affiliations:** Ludong University, Shandong Province, City Yantai, 264025 China

**Keywords:** Muscle aging, Exercise, High-salt diet, Heart, FOXO/SOD/PGC-1α

## Abstract

FOXO has long been associated with aging, exercise, and tissue homeostasis, but it remains unclear what the role is of the muscle FOXO gene in E against high-salt intake(HSI)-induced age-related defects of the skeletal muscle, heart, and mortality. In this research, overexpression and RNAi of the FOXO gene in the skeletal and heart muscle of *Drosophila* were constructed by building Mhc-GAL4/FOXO-UAS-overexpression and Mhc-GAL4/FOXO-UAS-RNAi system. The skeletal muscle and heart function, the balance of oxidation and antioxidant, and mitochondrial homeostasis were measured. The results showed that exercise reversed the age-related decline in climbing ability and downregulation of muscle FOXO expression induced by HSI. Muscle-specific FOXO-RNAi (FOXO-RNAi) and -overexpression (FOXO-OE) promoted or slowed the age-related decline in climbing ability, heart function, and skeletal muscle and heart structure damage, which was accompanied by the inhibition or activation of FOXO/PGC-1α/SDH and FOXO/SOD pathway activity, and oxidative stress (ROS) increased or decreased in both skeletal muscle and heart. The protective effect of exercise on the skeletal muscle and heart was blocked by FOXO-RNAi in aged HSI flies. FOXO-OE prolonged its lifespan, but it did not resist the HSI-induced lifespan shortening. Exercise did not improve HSI-induced lifespan shortening in FOXO-RNAi flies. Therefore, current results confirmed that the muscle FOXO gene played a vital role in exercise against age-related defects of the skeletal muscle and heart induced by HSI because it determined the activity of muscle FOXO/SOD and FOXO/PGC-1α/SDH pathways. The muscle FOXO gene also played an important role in exercise against HSI-induced mortality in aging flies.

## Introduction

In both humans and animals, the health of the skeletal and cardiac muscles is critical to the quality of life of older individuals. There is no doubt that premature aging of the skeletal muscle leads to an increased risk of skeletal muscle disease [1]. Age-related changes in the skeletal muscle can be accelerated by a high-salt diet. For example, sarcopenia is the main alteration occurring during aging, and HSI may lead to fat accumulation and muscle weakness associated with sarcopenia [2]. Moreover, HSI can lead to chronic comorbidities including hypertension, heart failure, and increase mortality [3]. However, exercise (E) delays the aging of the skeletal muscle, and it improves the quality of life of older individuals [4, 5]. Therefore, these pieces of evidence indicate that E and HSI have opposite effects on skeletal muscle aging, but the molecular mechanism of the interaction between E and HSI in age-related defects (ARD) of the skeletal muscle and heart is still poorly understood that.

Aging in the skeletal muscle and heart is accompanied by changes in many physiological factors, such as oxidative and antioxidant balance, mitochondrial homeostasis, and myofibrillar structure. FOXO regulates the aging by regulating these factors and shuttling from the cytoplasm to nucleus [6–13]. E can delay the aging, and the mechanism may be related to FOXO. For instance, it has been reported that moderate FOXO overexpression has cardioprotective effects and ameliorates non-pathological functional decline with age [14]. E improves heart function and delays heart aging by activating cardiac dSir2/FOXO pathways [15–18]. Therefore, these reports suggest that FOXO is involved in the exercise to delay myocardial and skeletal muscle aging, but it remains unknown whether FOXO plays a role in regulating E against HSI-induced ARD of the skeletal muscle and heart.

Extensive overexpression of body FOXO in flies can prevent HSI-induced ARD of the skeletal muscle and mortality [19, 20], but the role of muscle FOXO in exercise against HSI-induced ARD of mobility, heart function, and mortality remains unclear. In this research, overexpression and RNAi of the FOXO gene in the skeletal muscle and heart muscle of *Drosophila* were constructed by building Mhc-GAL4/FOXO-UAS- overexpression and Mhc-GAL4/FOXO-UAS-RNAi system. Then, the skeletal muscle function and heart function (climbing index, the time to fatigue, heart rate, fractional shortening, and so on), the balance of oxidation and antioxidant (SOD and ROS level), mitochondrial homeostasis (PGC-1α level, succinodehydrogenase (SDH), and myofibrils state (myofibrillary structure and Mhc level) were tested to further confirm whether FOXO in the skeletal muscle was involved in regulating E against ARD of the skeletal muscle and heart induced by HSI in *Drosophila*.

## Materials and methods

### Fly stocks, diet and husbandry, and exercise training protocols

The w^1118^ flies (stock ID: 3605; FlyBase Genotype: w^1118^), the *FOXO*-UAS-overexpression (*FOXO*-UAS-OE) flies (stock ID: 9575; FlyBase Genotype: y^1^ w^*^; P{UAS-foxo.P}2), and the MHC-gal4 (stock ID: 55,133; FlyBase Genotype: w^*^; P{Mhc-GAL4.K} 2/TM3, Sb^1^) flies were obtained from the Bloomington Stock Center. The *FOXO*-UAS-RNAi flies (stock ID: v106097; FlyBase Genotype: P{KK108590}VIE-260B) was obtained from the Vienna Drosophila Resource Center.

Normal food contained 1.6% soybean powder, 2.0% yeast, 6.7% corn meal, 0.7% agar, 4.8% sucrose, 4.8% maltose, and 0.3% propionic acid. Add 2% sodium chloride to normal food to make a high-salt food [19].

When constructing the exercise device, the advantage of the flies’ natural negative geotaxis behavior was taken to induce upward walking [21]. When flies climbed and reached the top of the vial, the top of the vial would be converted to the bottom by rotating the vial [22, 23]. Flies were exercised in the vial with an 8-cm length. Vials were rotated at the 60 rad/s. After the vials, each up-and-down turn, hold for 10 s for the flies to climb. Flies exercised for 1.5 h per day. They were trained for 5 days per week, and they rested on the other 2 days of the week.

All exercise groups, flies started exercise from when they were 2 days old and underwent a 5-week-long exercise program. All high-salt-intake flies started feeding high-salt food from when they were 2 days old and underwent a 5-week-long high-salt diet program.

### Climbing ability assay

The test vials for monitoring flies’ climbing speed and climbing failure were the same as the vials for exercise training. The test vials were left 8 cm in length for the flies to climb. There is a light box behind the vials once flies were shaken to the bottom of the vials, a timed digital camera snapped a picture after 3 s. The height of the fly climbs was clearly shown on the photographs. Seven pictures of each group were taken and averaged to arrive at a fixed height for each vial, and each vial contained about 20–23 flies.

A cohort of flies was observed during continuous stimulation by the exercise device. Flies were placed in the exercise device. Twenty flies were put into each vail. They were made to climb until fatigued. Fatigue was recognized as the failure that responded to a negative geotaxis stimulus with climbing behavior. The specific measurement protocol is referred to the study of Tinkerhess et al. [21].

### Heart function assay

Flies were anesthetized with FlyNap for 2–3 min. The head, ventral thorax, and ventral abdominal cuticle were removed by special glass needles in order to expose the heart and abdomen. Dissections were done in the oxygenated artificial hemolymph [24]. To get a random sampling of heart function, a single 30-s recording (AVI format) was made for each fly by using a high-speed camera. The heart physiology of the flies was assessed by using a AVS Video Editor analysis program that quantifies diastolic interval (DI), systolic interval (SI), heart period, heart rate, diastolic diameter, systolic diameter, and fractional shortening [25]. The sample size was 17 flies for each group.

### Lifespan assays

Dead flies were recorded daily. The lifespan was estimated for each fly as the number of days alive from eclosion to the day of death. The mean and median lifespan and survival curves were used to characterize the lifespan. The sample sizes were 200 to 210 flies per group [26].

### Transmission electron microscopy of the skeletal muscle and myocardium

According to the electron microscopic analysis, the skeletal muscle and myocardium were dissected in ice-cold fixative (2.5% glutaraldehyde in 0.1 M PIPES buffer at pH 7.4). After 10 h of fixation at 4 °C, the samples were washed with 0.1 M PIPES, post-fixed in 1% OsO_4_ (30 min), and stained in 2% uranyl acetate (1 h). The samples were dehydrated in an ethanol series (50%, 70%, 100%) and embedded in epoxy. Ultrathin Sects. (50 nm) were cut and viewed on a Tecnai G2 Spirit Bio-TWIN electron microscope [27].

### ELISA assay

The FOXO level, PGC-1α level, SDH level, SOD activity level, and ROS level were measured by ELISA assay (Insect FOXO, PGC-1α, SDH, SOD, and ROS ELISA Kits, MLBIO, Shanghai, China). Twenty flies’ muscles and 80 hearts were homogenized in PBS (pH 7.2–7.4). The samples were rapidly frozen in liquid nitrogen and then maintained at 2–8 °C after melting. Homogenize the samples with grinders, and centrifugation was conducted for 20 min at 2000–3000 rpm. Then, we removed the supernatant. Assay: take the blank well as zero, and read absorbance at 450 nm within 15 min of adding Stop Solution.

### qRT-PCR

About 20 flies’ muscles and 80 hearts were homogenized in Trizol. 10 μg of the total RNA was purified by organic solvent extraction from the Trizol (TRIzol, Invitrogen). The purified RNA was treated with DNase I (RNase-free, Roche), and it was used to produce oligo dT-primed cDNAs (SuperScript II RT, Invitrogen), which were then used as templates for quantitative real-time PCR. The rp49 gene was used as an internal reference for normalizing the quantity of total RNAs. The real-time PCR was performed with SYBR green using an ABI7300 Real-time PCR Instrument (Applied Biosystems), with 3 biological replicates. Expression of the various genes was determined by the comparative CT method (ABI Prism 7700 Sequence Detection System User Bulletin #2, Applied Biosystems). Primer sequences of FOXO were as follows: F: 5′-AACAACAGCAGCATCAGCAG-3′; R: 5′-CTGAACC CGAGCATTCAGAT-3′. Primer sequences of PGC-1α were as follows: F: 5′-TGTTGCTGCTACTGCTGCTT-3′; R: 5′-GCCTC TGCATCACCTACACA-3′. Primer sequences of Mhc were as follows: F: 5′-TGCGTTGCCATCAATCCT-3′; R: 5′-GTAGGCAC CGTCAGAGATGG-3′. Primer sequences of Rp49 were as follows: F: 5 -CTAAGCTG TCGCACAAATGG-3′; R: 5′-AACTT CTTGAATCC GGTGGG-3′.

### Statistical analyses

Independent-sample tests were used to assess differences between the 1-week-old flies and the 7-week-old flies. The 1-way analysis of variance (ANOVA) with least significant difference (LSD) tests was used to identify differences among these groups. *P* values for lifespan curves and climbing endurance curves were calculated by the log-rank test. Analyses were performed using the Statistical Package for the Social Sciences (SPSS) version 16.0 for Windows (SPSS Inc., Chicago, USA), with a statistical significance set at *P* < 0.05. Data are represented as means ± SEM.

## Results

### Exercise ameliorates locomotor impairment and FOXO expression downregulation induced by HSI in the skeletal muscle in aging Drosophila

Our previous studies have been confirmed that HSI caused premature aging in w^1118^ flies. The system overexpression of FOXO in flies can prevent HSI-induced premature aging of the skeletal muscle. The increased FOXO/SOD pathway activity plays an important role in mediating E resistance to HSI-induced impairment of climbing capacity, heart defects, and longevity in aging *Drosophila* [19, 20]. However, the role of the muscle FOXO gene in E against HSI-induced premature aging of the skeletal muscle remains unclear.

**Fig. 1:**
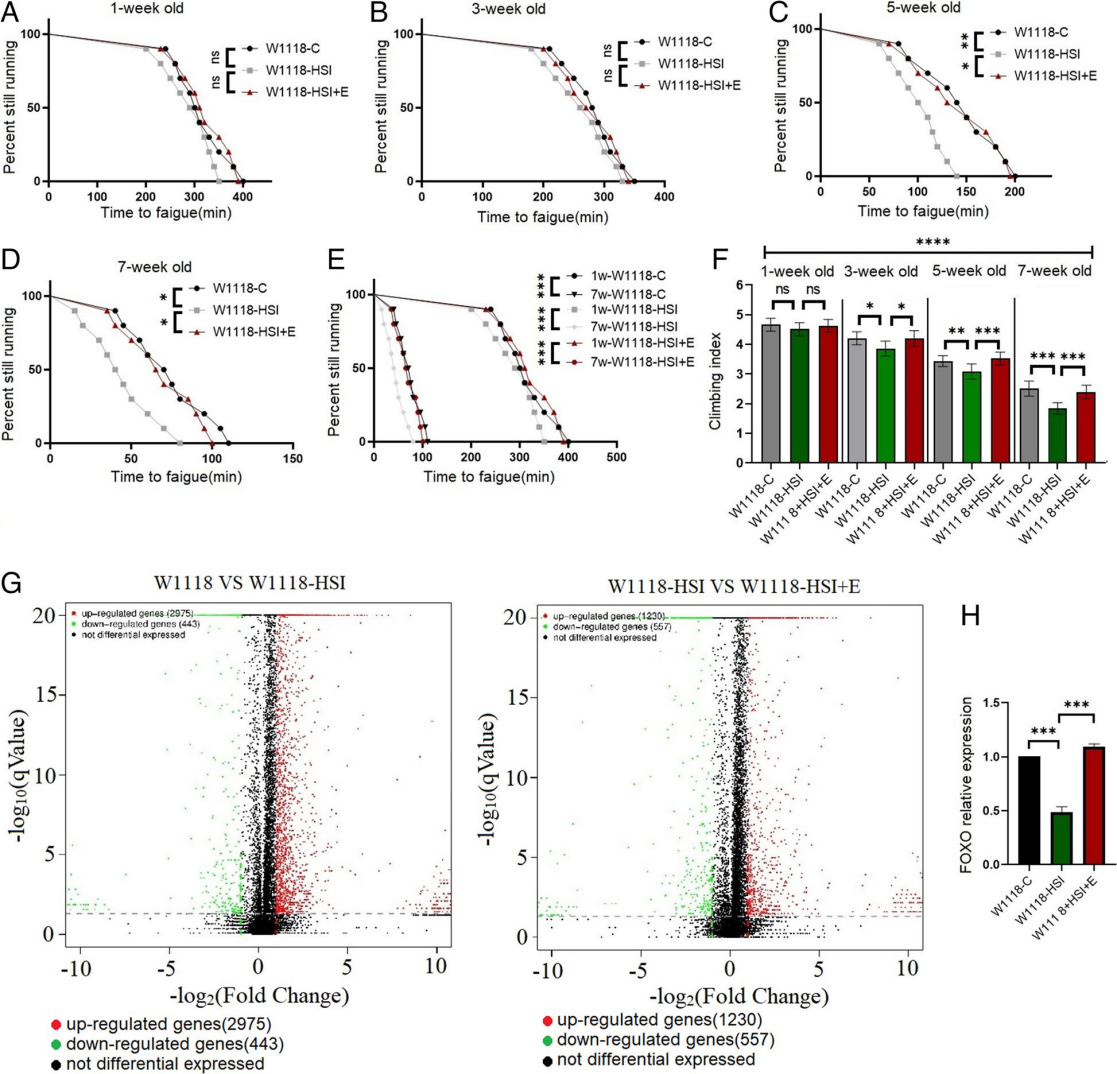
The effect of HSI and exercise combine with HSI on climbing endurance and rapid climbing ability in w^1118^ flies. **A** Time to fatigue in 1-week-old flies. **B** Time to fatigue in 3-week-old flies. **C** Time to fatigue in 5-week-old flies. **D** Time to fatigue in 7-week-old flies. **E** Time to fatigue changed with age in flies. **F** Climbing index in 3 s at different ages. **G–I** FOXO expression results of high-throughput sequencing and qRT-PCR in the skeletal muscle, L-1: w^1118^ vs w^1118^-HSI; L-2: w^1118^-HSI vs w^1118^-HSI + E. For climbing endurance, the sample size was 200–220 flies for each group. *P* values for climbing endurance curves were calculated by the log-rank test. For climbing index measurement, the sample size was about 150–170 flies for each group. The one-way analysis of variance (ANOVA) with least significant difference (LSD) tests was used to identify differences among the groups. Data are represented as means ± SEM. *P < 0.05; **P < 0.01; ***P < 0.001; ns means no significant differences

In this study, we first screened and determined whether the FOXO gene of the skeletal muscle was involved in E against HSI-induced climbing ability decline in w^1118^ wild flies. The results showed that at the age of 5 and 7 weeks, HSI significantly decreased the time to fatigue (TTF) and climbing index (CI) of w^1118^ flies (*P* < 0.05 or *P* < 0.01) (Fig. 1C, D, and F). At the age of 5 and 7 weeks, E significantly increased the TTF and CI of w^1118^-HSI flies (*P* < 0.05 or *P* < 0.01) (Fig. 1C, D, and F). At the age of 1 and 3 weeks, both HSI and E combine with HSI did not significantly change the CI and TTF of w^1118^ flies (*P* > 0.05) (Fig. 1A, B, and F). Aging significantly decreased the TTF and CI of w^1118^ flies (*P* < 0.001) (Fig. 1E and F). The results of high-throughput sequencing and qRT-PCR showed that at the age of 5 weeks, HSI significantly downregulated the relative FOXO expression of the skeletal muscle in flies (*P* < 0.01), but E significantly upregulated the relative FOXO expression of the skeletal muscle in HSI flies (*P* < 0.01) (Fig. 1G and H).

These results suggested that the muscle FOXO gene was involved in E against HSI-induced ARD of the skeletal muscle in aging w^1118^ Drosophila, but the role of the muscle FOXO gene in E against HSI-induced ARD of the skeletal muscle still remained unclear.

### Role of FOXO-RNAi in exercise against the skeletal muscle and cardiac ARD caused by high-salt intake

To further confirm the role of muscle FOXO in E against HSI-induced premature aging of the skeletal muscle, we then conducted E and HSI intervention in FOXO-OE flies and FOXO-RNAi flies. It has been confirmed that FOXO overexpression has cardioprotective effects and ameliorates non-pathological functional decline with age [14], but the role of E against HSI-induced ARD of the heart is still poorly understood.

**Fig. 2:**
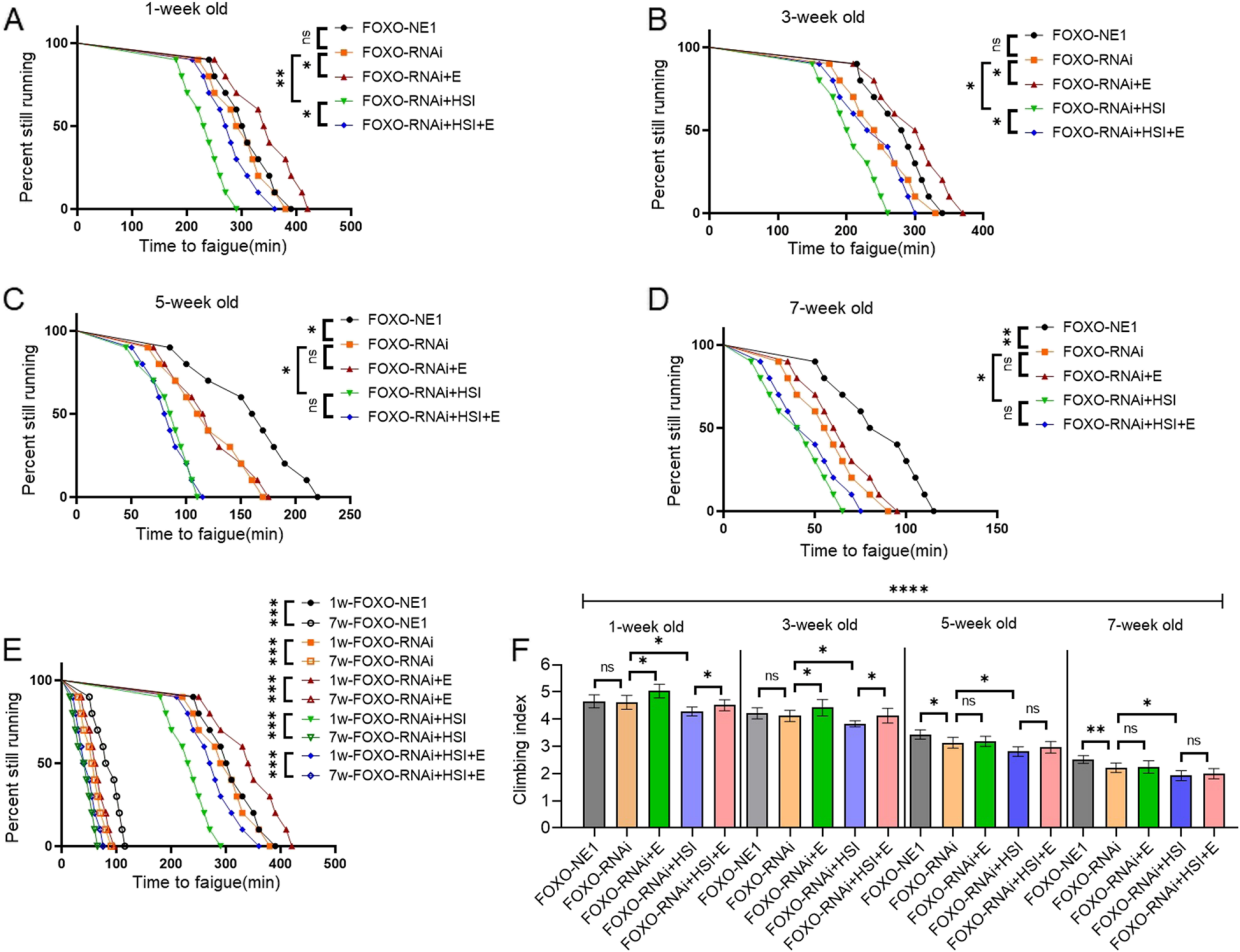
The effect of HSI, exercise, and FOXO-RNAi on climbing endurance and rapid climbing ability in flies. **A** Time to fatigue in 1-week-old and FOXO-RNAi flies. **B** Time to fatigue in 3-week-old and FOXO-RNAi flies. **C** Time to fatigue in 5-week-old and FOXO-RNAi flies. **D** Time to fatigue in 7-week-old and FOXO-RNAi flies. **E** Time to fatigue changed with age in FOXO-RNAi flies. **F** Climbing index in 3 s at different ages. For climbing endurance, the sample size was 200–220 flies for each group. *P* values for climbing endurance curves were calculated by the log-rank test. For climbing index measurement, the sample size was about 150–170 flies for each group. The 1-way analysis of variance (ANOVA) with least significant difference (LSD) tests was used to identify differences among the groups. Data are represented as means ± SEM. *P < 0.05; **P < 0.01; ***P < 0.001; ns means no significant differences. Male MHC-gal4 flies were crossed to the female *FOXO*-UAS-RNAi line. “P{KK108590} VIE-260B” and “P{KK108590}VIE-260B > MHC-gal4” were represented as “FOXO-normal-expression-1(FOXO-NE1)” and “FOXO-RNAi,” respectively

The results showed that at the age of 5 and 7 weeks, FOXO-RNAi significantly decreased the TTF of flies (*P* < 0.05, *P* < 0.01) (Fig. 2C, D) and the CI of flies (*P* < 0.05) (Fig. 2F). At the age of 1 and 3 weeks, FOXO-RNAi did not significantly change the CI and TTF (*P* > 0.05) (Fig. 2A, B, and F). Aging significantly decreased the TTF and CI of FOXO-NE1 and FOXO-RNAi-C flies (*P* < 0.001) (Fig. 2E). At the age of 1, 3, 5, and 7 weeks, HSI significantly decreased the TTF of FOXO-RNAi flies (*P* < 0.05 or *P* < 0.01) (Fig. 2A, D) and the CI of FOXO-RNAi flies (*P* < 0.05) (Fig. 2F). Aging significantly decreased the TTF and CI of FOXO-RNAi + HSI flies (*P* < 0.001) (Fig. 2E and F). At the age of 1 and 3 weeks, E significantly increased the TTF and CI of FOXO-RNAi-HSI flies (*P* < 0.05) (Fig. 2A, B), but at the age of 5 and 7 weeks, E did not significantly change the TTF and CI of FOXO-RNAi-HSI flies (*P* > 0.05) (Fig. 2C, D). Aging significantly decreased the TTF and CI of FOXO-RNAi + HIS + E flies (*P* < 0.001) (Fig. 2E, F).

**Fig. 3:**
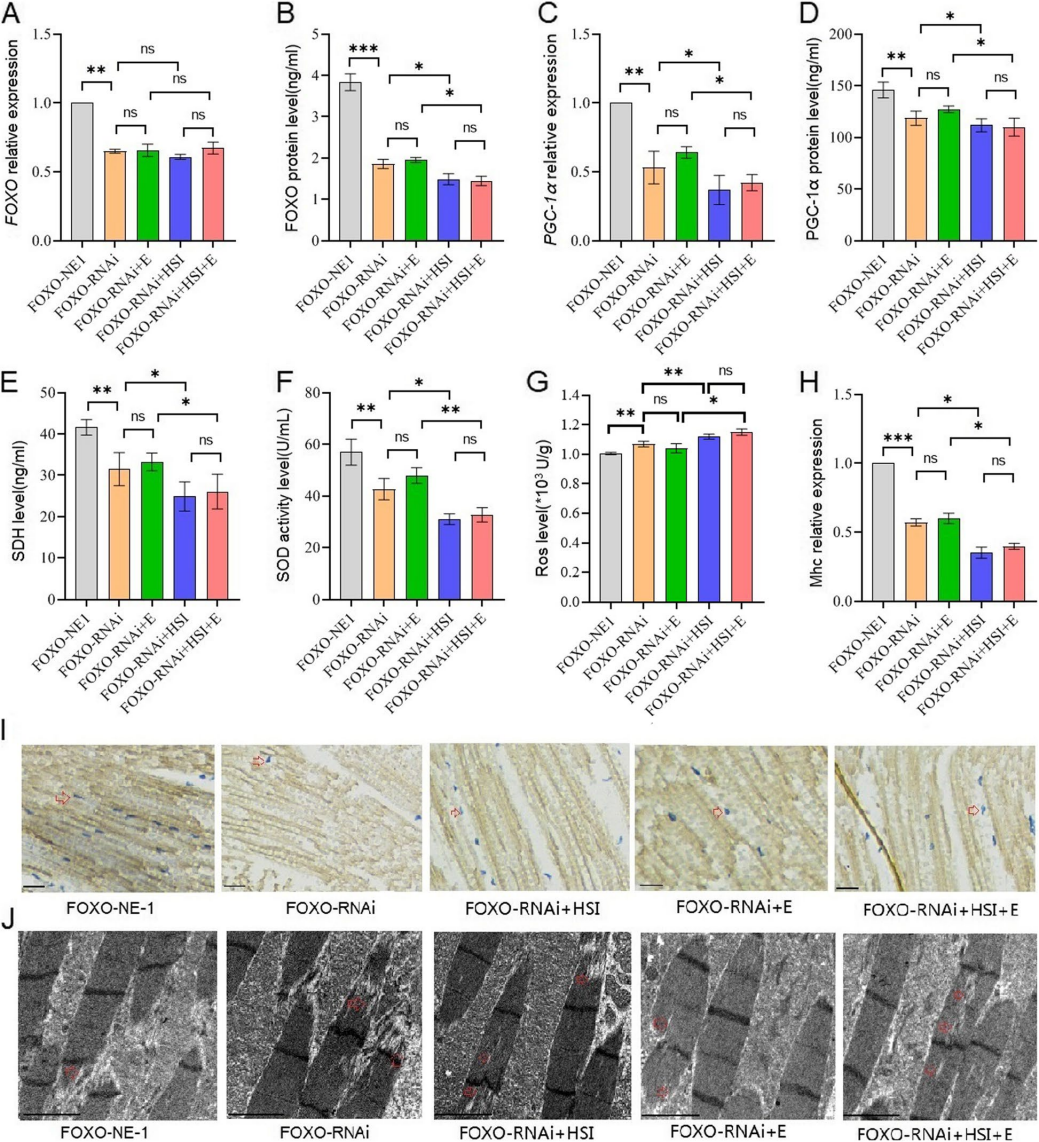
The effect of HSI, exercise, and FOXO-RNAi on the skeletal muscle physiology and structure in 5-week-old flies. **A** The skeletal muscle relative *FOXO* expression. **B** The skeletal muscle relative FOXO protein level. **C** The skeletal muscle relative PGC-1α expression. **D** The skeletal muscle PGC-1α protein level. **E** The skeletal muscle succinodehydrogenase (SDH) protein level. **F** The skeletal muscle SOD activity levels. **G** The skeletal muscle ROS level. **H** The skeletal muscle myosin heavy chain (Mhc) expression level. **I** Images of MHC immunohistochemistry in the skeletal muscle (scale: the black line represents 10 microns, the red arrow is the nucleus, and the yellow structure represents muscle cells). **J** Transmission electron microscopy of the skeletal muscle (scale: the black line represents 2 microns). The red arrow indicates the myofibrillary injury. For proteins and ROS, the sample size was 20 flies’ muscles for each group, and measurements were taken 3 times. For RT-PCR and ELISA, the sample size was 20 flies’ muscles for each group, and measurements were taken 3 times. The 1-way analysis of variance (ANOVA) with least significant difference (LSD) tests was used to identify differences among the groups. Data are represented as means ± SEM. *P < 0.05; **P < 0.01; ns means no significant differences

Besides, FOXO-RNAi significantly decreased the FOXO gene expression and protein level, PGC-1α expression and protein levels, SDH protein level, SOD activity level, and Mhc expression level, and it significantly increased ROS level of the skeletal muscle (*P* < 0.05 or *P* < 0.01) (Fig. 3A to I). Moreover, the transmission electron microscope images displayed that FOXO-RNAi might increase myofibrillary damage caused by aging (Fig. 3J). Finally, in aged FOXO-RNAi flies, HSI significantly decreased the FOXO protein level, PGC-1α expression and protein levels, SDH protein level, SOD activity level, and Mhc expression level, and it significantly increased the ROS level of the skeletal muscle (*P* < 0.05 or *P* < 0.01) (Fig. 3A to I). Moreover, the transmission electron microscope images displayed that HSI might further increase myofibrillary damage caused by aging in FOXO-RNAi flies (Fig. 3J). In additon, in aged FOXO-RNAi-HSI flies, E also did not significantly change the FOXO gene expression and protein level, PGC-1α expression and protein levels, SDH protein level, SOD activity level, MHC expression level, and ROS level of the skeletal muscle (*P* > 0.05) (Fig. 3A to I). Moreover, the transmission electron microscope images displayed that E might not decrease myofibrillary damage caused by aging in FOXO-RNAi-HSI flies (Fig. 3J).

**Fig. 4:**
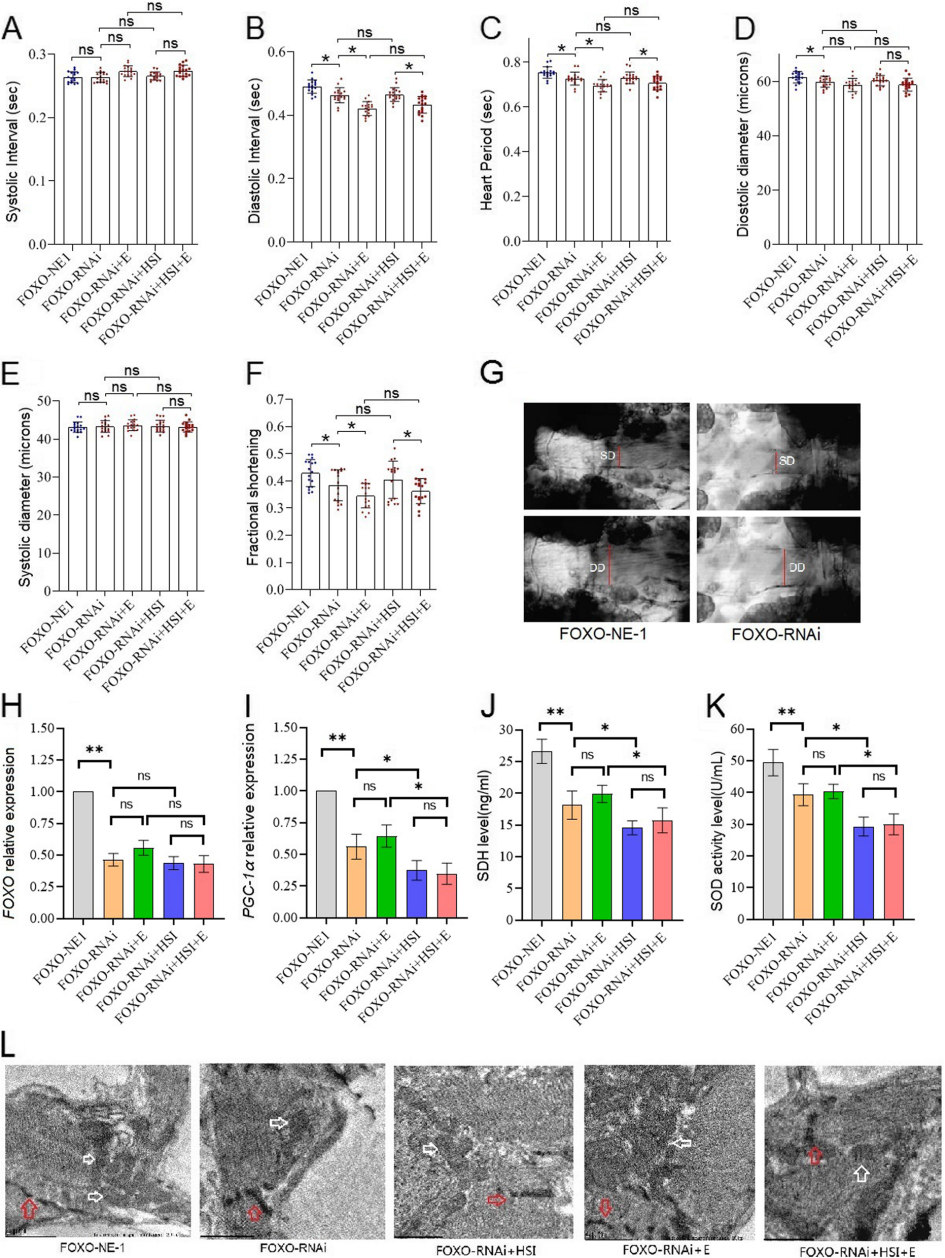
The effect of HSI, exercise, and FOXO-RNAi on heart function, physiology, and structure in 5-week-old flies. **A** Heart systolic period. **B** Heart diastolic period. **C** Heart period. **D** Systolic diameter. **E** Diastolic diameter. **F** Fractional shortening. **G** Systolic image and diastolic image. SD, systolic diameter; DD, diastolic diameter. **H** Heart relative *FOXO* expression. **I** Heart relative PGC-1α expression. **J** Heart succinodehydrogenase (SDH) protein level. **K** Heart SOD activity levels. **L** Transmission electron microscopy of hearts (scale: the black line represents 1 micron). The red arrows indicate the myofibrillary and Z line. The white arrows indicate the mitochondria. FOXO-RNAi and HSI could damage myofibrils and mitochondria, and FOXO-RNAi could block the protective effect of exercise on myofibrils and mitochondria. FOXO-OE and exercise could protect myofibrils and mitochondria from HSI damage. For heart function, the sample size was 22 hearts for each group. For heart failure, the sample size was 30 hearts for each group. For RT-PCR and ELISA, the sample size was 80 flies’ hearts for each group, and measurements were taken 3 times. The 1-way analysis of variance (ANOVA) with least significant difference (LSD) tests was used to identify differences among the groups. Data are represented as means ± SEM. *P < 0.05; **P < 0.01; ns means no significant differences

FOXO-RNAi significantly reduced cardiac diastolic interval (DI), heart period (HP), diastolic diameter (DM), fractional shortening (FS), FOXO expression level, PGC-1α expression level, SDH protein level, and SOD activity level (*P* < 0.05 or *P* < 0.01) (Fig. 4A to K). Moreover, the transmission electron microscope images displayed that FOXO-RNAi reduced mitochondria in cardiomyocytes in old flies (Fig. 4L). In order to confirm the role of the cardiac *FOXO* gene in HSI-induced PA of the heart, a high-salt diet intervention was conducted in flies with specific regulation of myocardial *FOXO* expression. The results showed that HSI significantly reduced cardiac DI, HP, FS, and FOXO expression level, PGC-1α expression level, SDH protein level, and SOD activity level in old FOXO-RNAi flies (*P* < 0.05 or *P* < 0.01) (Fig. 4A to K). Moreover, the transmission electron microscope images displayed that HSI-reduced mitochondria of cardiomyocytes in old FOXO-RNAi flies (Fig. 4L). To confirm the role of the cardiac *FOXO* gene in E against PA in the heart, E intervention was performed in flies with specific regulation of myocardial FOXO expression. The results showed that in old FOXO-RNAi flies, E did not significantly change cardiac DI, HP, FS, and FOXO expression level; PGC-1α expression level; SDH protein level; and SOD activity level (*P* > 0.05) (Fig. 4A to K). Moreover, the transmission electron microscope images displayed that E did not significantly have mitochondria of cardiomyocytes in old FOXO-RNAi flies (Fig. 4L).

These results confirmed that the improvement of E in HSI-induced ARD of the skeletal muscle and heart can be blocked by FOXO-RNAi through inhibiting their FOXO/SOD and FOXO/PGC-1α/SDH pathways.

### The improvement of E in HSI-induced ARD of the skeletal muscle and heart can be further enhanced by FOXO-OE

To further confirm the role of the muscle FOXO gene in E against ARD induced by HSI, FOXO-OE flies were subjected to E and HSI interventions.

**Fig. 5:**
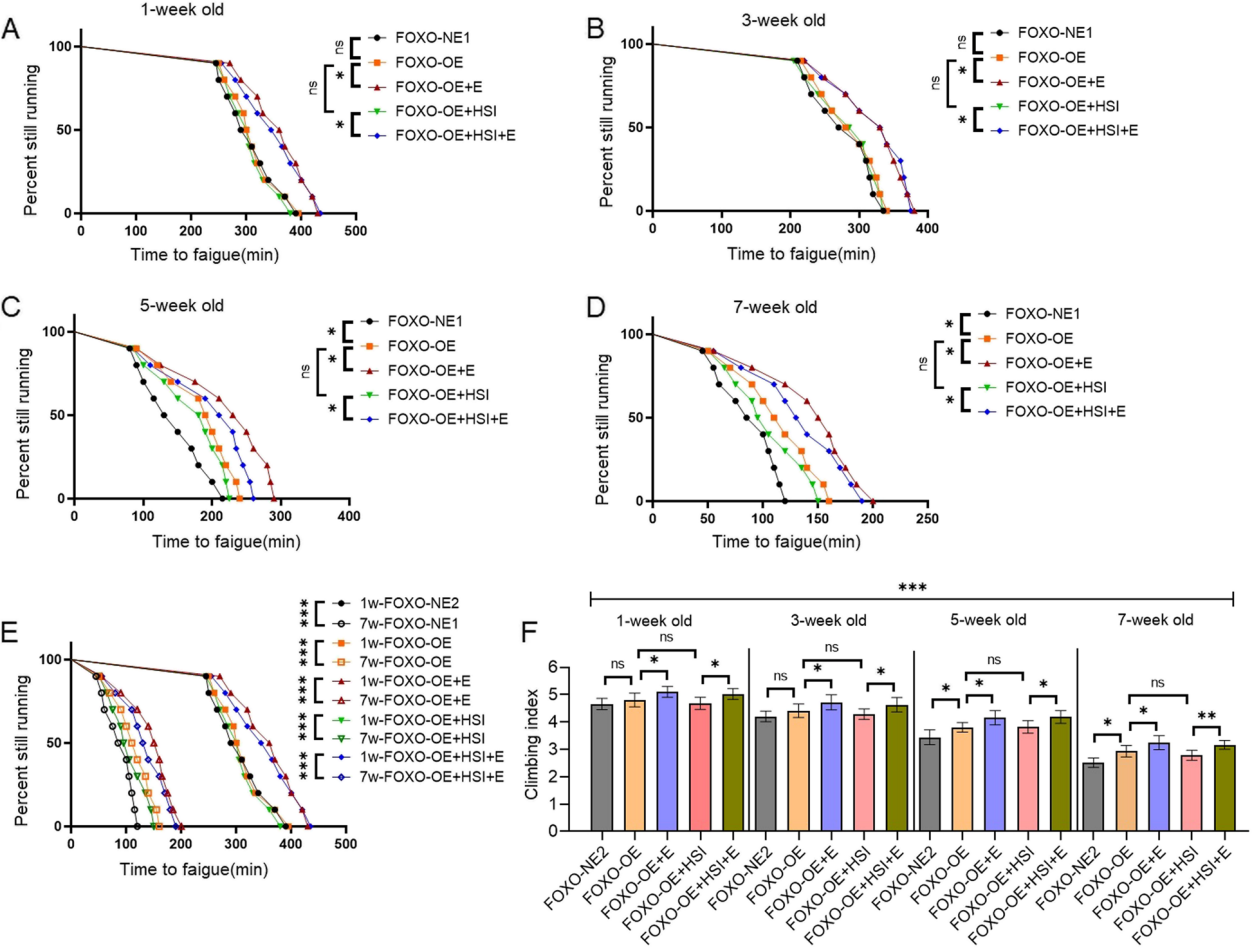
The effect of HSI, exercise, and FOXO-OE on climbing endurance and rapid climbing ability in flies. **A** Time to fatigue in 1-week-old and FOXO-OE flies. **B** Time to fatigue in 3-week-old and FOXO-OE flies. **C** Time to fatigue in 5-week-old and FOXO-OE flies. **D** Time to fatigue in 7-week-old and FOXO-OE flies. **E** Time to fatigue changed with age in FOXO-OE flies. **F** Climbing index in 3 s at different ages. For climbing endurance, the sample size was 200–220 flies for each group. *P* values for climbing endurance curves were calculated by the log-rank test. For climbing index measurement, the sample size was about 150–170 flies for each group. The 1-way analysis of variance (ANOVA) with least significant difference (LSD) tests was used to identify differences among the groups. Data are represented as means ± SEM. *P < 0.05, **P < 0.01, ***P < 0.001; ns means no significant differences. Male MHC-gal4 flies were crossed to female *FOXO*-UAS-OE line a. “P{UAS-foxo.P}2 > MHC-gal4” and P{UAS-foxo.P}2 > MHC-gal4″ were represented as “FOXO-normal-expression-2 (FOXO-NE2)” and “FOXO-overexpression (FOXO-OE),” respectively

The results showed that at the age of 5 and 7 weeks, FOXO-OE significantly increased the TTF (*P* < 0.05) (Fig. 5C, D) and the CI of flies (*P* < 0.05) (Fig. 5F). At the age of 1 and 3 weeks, FOXO-OE did not significantly change the CI and TTF (*P* > 0.05) (Fig. 5A, B, and F). Aging significantly decreased the TTF and CI of FOXO-NE2 and FOXO-OE flies (*P* < 0.001) (Fig. 5E). Moreover, at the age of 1, 3, 5, and 7 weeks, HSI did not significantly change the TTF and CI of FOXO-OE flies (*P* > 0.05) (Fig. 5A to D, F). Aging significantly decreased the TTF and CI of FOXO-OE + HSI flies (*P* < 0.001) (Fig. 5E and F). At the age of 1, 3, 5, and 7 weeks, E further significantly increased the TTF of FOXO-OE flies (*P* < 0.05 or *P* < 0.01) (Fig. 5A to D) and the CI of FOXO-OE flies (*P* < 0.05) (Fig. 5F). Aging significantly decreased the TTF and CI of FOXO-OE + E flies (*P* < 0.001) (Fig. 5E and F). At the age of 1, 3, 5, and 7 weeks, E significantly increased the TTF of FOXO-OE-HSI flies (*P* < 0.05) (Fig. 5A to D), and it also significantly increased the CI of FOXO-OE-HSI flies at the age of 1, 3, 5, and 7 weeks (*P* < 0.05) (Fig. 5F). Aging significantly decreased the TTF and CI of FOXO-OE + HIS + E flies (*P* < 0.001) (Fig. 5E and F).

**Fig. 6:**
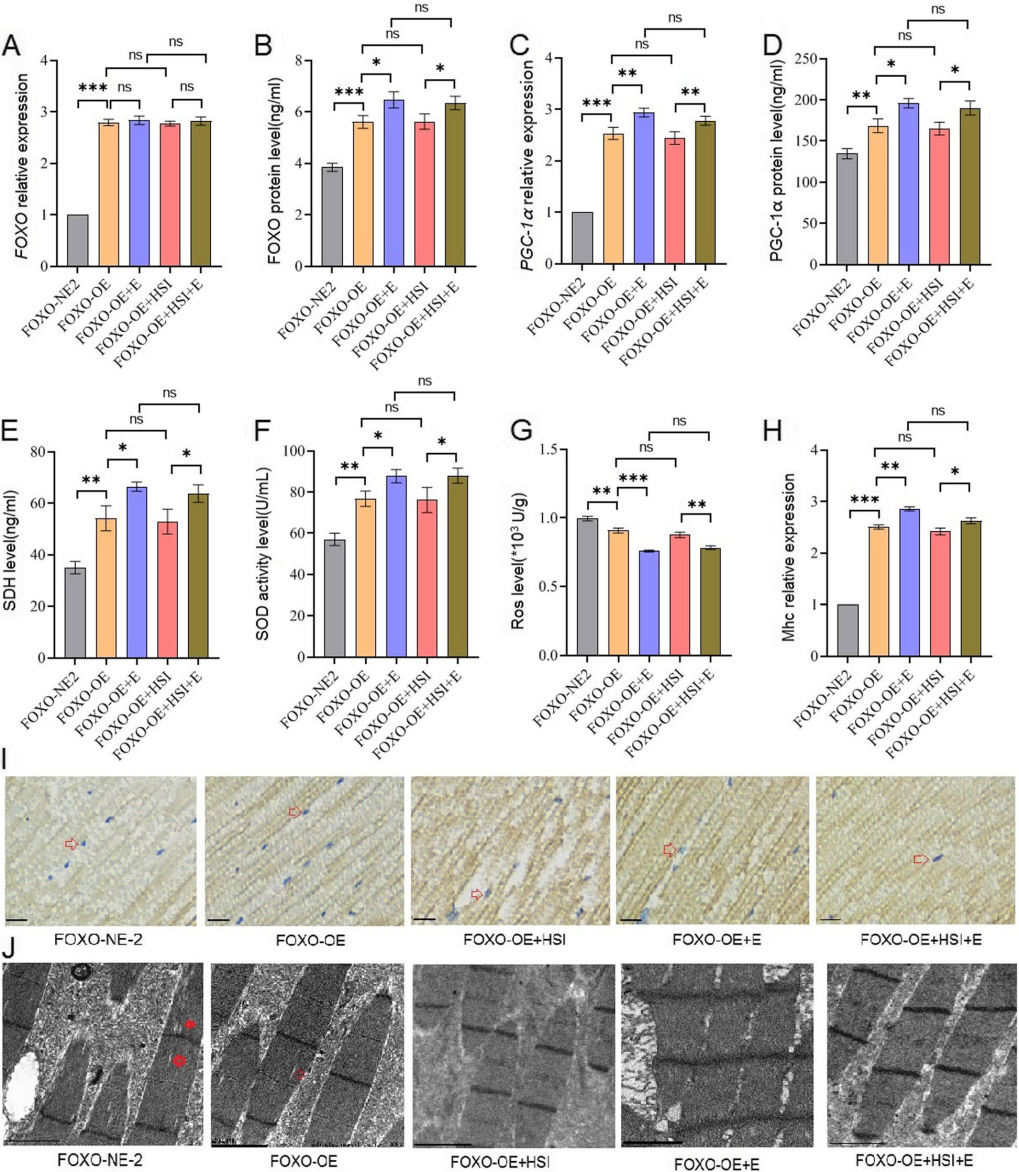
The effect of HSI, exercise, and FOXO-OE on the skeletal muscle physiology and structure in 5-week-old flies. **A** The skeletal muscle relative *FOXO* expression. **B** The skeletal muscle relative FOXO protein level. **C** The skeletal muscle relative PGC-1α expression. **D** The skeletal muscle PGC-1α protein level. **E** The skeletal muscle succinodehydrogenase (SDH) protein level. **F** The skeletal muscle SOD activity levels. **G** The skeletal muscle ROS level. **H** The skeletal muscle myosin heavy chain (Mhc) expression level. **I** Images of MHC immunohistochemistry in the skeletal muscle (scale: the black line represents 10 microns, the red arrow is the nucleus, and the yellow structure represents muscle cells). **J** Transmission electron microscopy of the skeletal muscle (scale: the black line represents 2 microns). The red arrow indicates the myofibrillary injury. For proteins and ROS, the sample size was 20 flies’ muscles for each group, and measurements were taken 3 times. For RT-PCR and ELISA, the sample size was 20 flies’ muscles for each group, and measurements were taken 3 times. The 1-way analysis of variance (ANOVA) with least significant difference (LSD) tests was used to identify differences among the groups. Data are represented as means ± SEM. *P < 0.05, **P < 0.01; ns means no significant differences

FOXO-OE significantly increased the FOXO gene expression and protein level, PGC-1α expression and protein levels, SDH protein level, SOD activity level, and Mhc expression level. It significantly decreased the ROS level of the skeletal muscle (*P* < 0.05 or *P* < 0.01) (Fig. 6A to I). Moreover, the transmission electron microscope images displayed that FOXO-OE might reduce myofibrillary damage caused by aging (Fig. 6J). Besides, in the aged FOXO-OE flies, HSI also did not significantly change the FOXO gene expression and protein level, PGC-1α expression and protein levels, SDH protein level, SOD activity level, Mhc expression level, and ROS level of the skeletal muscle (*P* > 0.05) (Fig. 6A to I). Moreover, the transmission electron microscope images displayed that HSI might not increase myofibrillary damage caused by aging in FOXO-OE flies (Fig. 6J). What is more, E further significantly increased the FOXO protein level, PGC-1α expression and protein levels, SDH protein level, SOD activity level, and MHC expression level. And it also further significantly decreased the ROS level of the skeletal muscle in FOXO-OE flies (*P* < 0.05 or *P* < 0.01) (Fig. 6A to I). Moreover, the transmission electron microscope images displayed that E might protect myofibrillary from damage caused by aging (Fig. 6J). Furthermore, in aged FOXO-OE-HSI flies, E significantly increased the FOXO expression and protein level, PGC-1α expression and protein levels, SDH protein level, SOD activity level, and Mhc expression level. It significantly decreased the ROS level of the skeletal muscle (*P* < 0.05 or *P* < 0.01) (Fig. 6A to I). Moreover, the transmission electron microscope images displayed that E might decrease myofibrillary damage caused by aging in FOXO-OE-HSI flies (Fig. 6J).

**Fig. 7:**
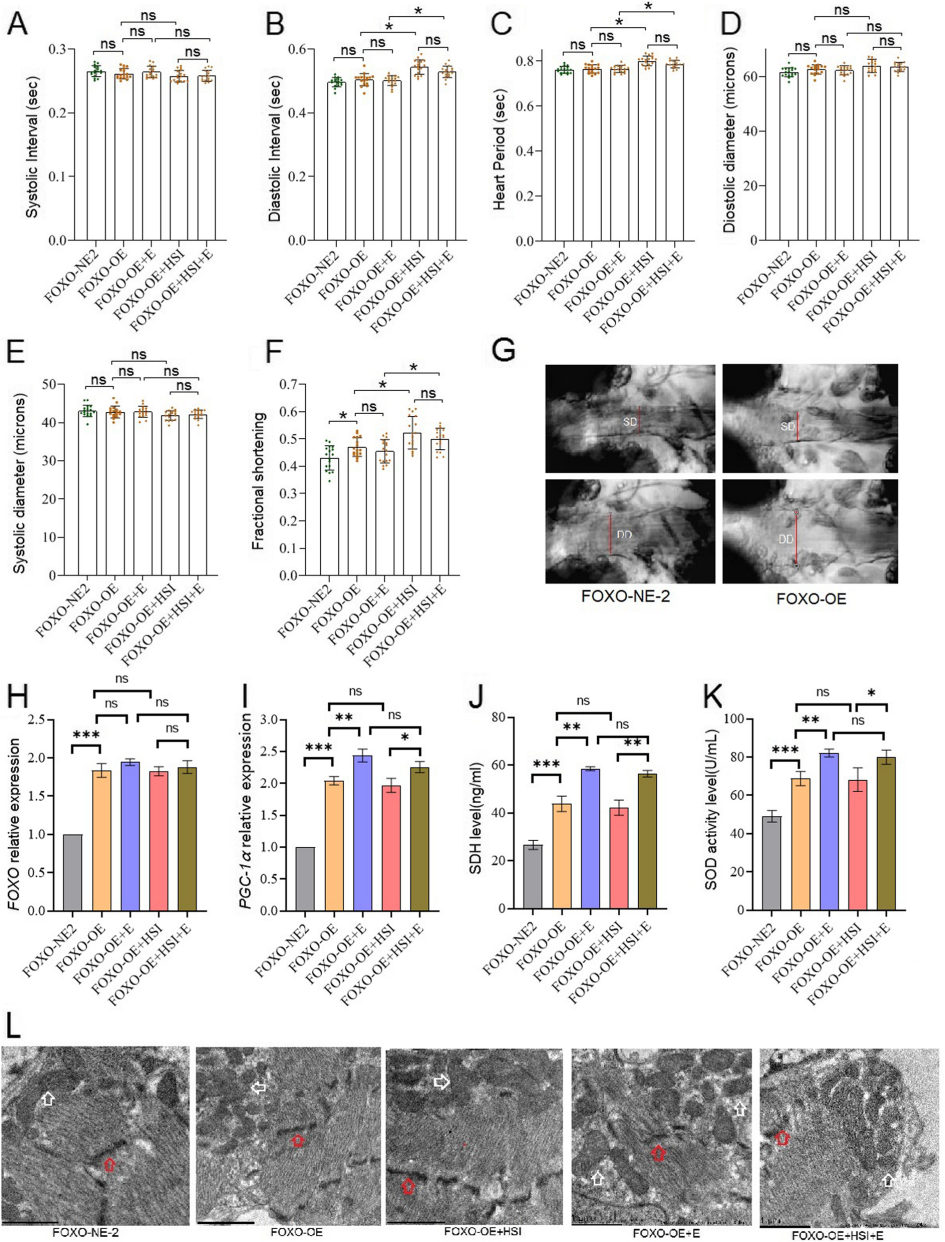
The effect of HSI, exercise, and FOXO-OE on heart function, physiology, and structure in 5-week-old flies. **A** Heart systolic period. **B** Heart diastolic period. **C** Heart period. **D** Systolic diameter. **E** Diastolic diameter. **F** Fractional shortening. **G** Systolic image and diastolic image. SD, systolic diameter; DD, diastolic diameter. **H** Heart relative *FOXO* expression. **I** Heart relative *PGC-1α* expression. **J** Heart succinodehydrogenase (SDH) protein level. **K** Heart SOD activity levels. **L** Transmission electron microscopy of hearts (scale: the black line represents 1 micron). The red arrows indicate the myofibrillary and Z line. The white arrows indicate the mitochondria. FOXO-RNAi and HSI could damage myofibrils and mitochondria, and FOXO-RNAi could block the protective effect of exercise on myofibrils and mitochondria. FOXO-OE and exercise could protect myofibrils and mitochondria from HSI damage. For heart function, the sample size was 22 hearts for each group. For heart failure, the sample size was 30 hearts for each group. For RT-PCR and ELISA, the sample size was 80 flies’ hearts for each group, and measurements were taken 3 times. The 1-way analysis of variance (ANOVA) with least significant difference (LSD) tests was used to identify differences among the groups. Data are represented as means ± SEM. *P < 0.05, **P < 0.01; ns means no significant differences

FOXO-OE significantly increased the FS, FOXO expression level, PGC-1α expression level, SDH protein level, and SOD activity level (*P* < 0.05 or *P* < 0.01) (Fig. 7A to K). In addition, the transmission electron microscope images displayed that FOXO-OE increased mitochondria in cardiomyocytes in old flies (Fig. 7L). In old FOXO-OE flies, HSI did not significantly change cardiac DI, HP, FS, and FOXO expression level; PGC-1α expression level; SDH protein level; and SOD activity level (*P* > 0.05) (Fig. 7A to K). Moreover, the transmission electron microscope images displayed that HSI did not significantly change the mitochondria of cardiomyocytes in old FOXO-OE flies (Fig. 7L). In old FOXO-OE flies, E significantly increased cardiac DI, HP, FS, and FOXO expression level; PGC-1α expression level; SDH protein level; and SOD activity level in old FOXO-RNAi flies (*P* < 0.05 or *P* < 0.01) (Fig. 7A to K). Moreover, the transmission electron microscope images displayed that E increased mitochondria of cardiomyocytes in old FOXO-OE flies (Fig. 7L). In old FOXO-OE HSI flies, E significantly reduced cardiac DI, HP, FS, and FOXO expression level; PGC-1α expression level; SDH protein level; and SOD activity level in old FOXO-RNAi flies (*P* < 0.05 or *P* < 0.01) (Fig. 7A to K). Moreover, the transmission electron microscope images displayed that E increased mitochondria of cardiomyocytes in old FOXO-OE HSI flies (Fig. 7L).

These results confirmed that the improvement of E in HSI-induced ARD of the skeletal muscle and heart can be further enhanced by FOXO-OE through further activating their FOXO/SOD and FOXO/PGC-1α/SDH pathways.

### The effect of E, HSI, FOXO-RNAi, and FOXO-OE on lifespan in Drosophila

**Fig. 8:**
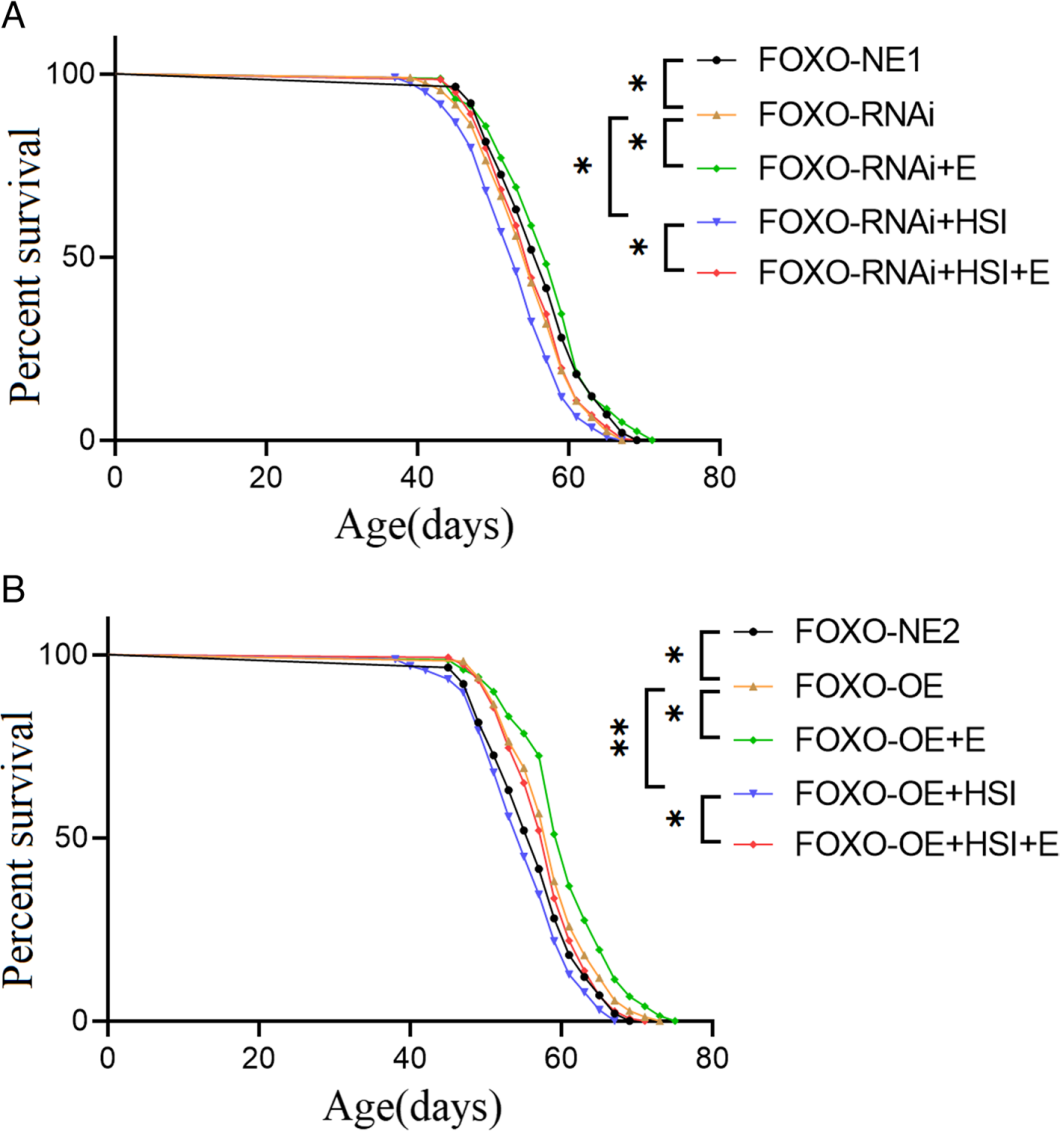
The effect of HSI, exercise, FOXO-RNAi, and FOXO-OE on lifespan. *P* values for lifespan curves were calculated by the log-rank test

The results showed that the lifespan of FOXO-RNAi flies was significantly shorter than that of FOXO-NE1 flies (*P* < 0.01). The lifespan of FOXO-RNAi + HSI flies was significantly shorter than that of FOXO-RNAi flies (*P* < 0.001). The lifespan of FOXO-RNAi + HSI flies significantly increased after exercise training (*P* > 0.05) (Fig. 8A). Moreover, the lifespan of FOXO-OE flies was significantly longer than that of FOXO-NE2 flies (*P* < 0.05). The lifespan of FOXO-OE + HSI flies was significantly shorter than that of FOXO-OE flies (*P* < 0.001). The lifespan of FOXO-OE + HSI + E flies was significantly longer than that of FOXO-OE + HSI flies (*P* < 0.05) (Fig. 8B).

These results suggested that FOXO-OE prolonged the lifespan of flies, but it did not resist the HSI-induced lifespan shortening. E combined with overexpression of FOXO in muscle improved the longevity in HSI flies. FOXO-RNAi decreased the lifespan of flies, and it further decreased longevity when combined with HSI. E improved HSI-induced lifespan shortening in FOXO-RNAi flies. Therefore, the muscle FOXO gene played an important role in E against HSI-induced lifespan shortening.

## Discussion

In flies, overexpression of *the* FOXO gene seems to delay the aging process. For example, alpha-ketoglutarate can extend lifespan in flies remarkably by upregulating mRNA expression of FOXO, Sirt1, and AMPKα [28]. Korean mistletoe also extends the lifespan via FOXO activation induced by dSir2 in *Drosophila* [29]. Moreover, Zoledronate extends lifespan, improves climbing activity, and reduces intestinal epithelial dysplasia and permeability with age by upregulating FOXO activation [30]. Moreover, Minocycline-induced lifespan extension is associated with increased resistance to an oxidative stressor, and minocycline’s effects on lifespan and resistance to oxidative stress are largely abrogated in FOXO null mutant, and the drug treatment increases the activity of FOXO [31]. Phosphoglycerate Mutase 5 promotes lasting FOXO activation after developmental mitochondrial stress and extends the lifespan in Drosophila [32]. Next, overexpression of FOXO is cardioprotective so that it can ameliorate nonpathological functional decline with age. Conversely, excessive FOXO overexpression or suppression proves detrimental to heart function and/or organismal development [14]. Furthermore, FOXO/4E-BP signaling in muscles decreases feeding behavior and the release of insulin from producing cells, which in turn delays the age-related accumulation of protein aggregates in other tissues [33]. Motor neuron-specific overexpression of FOXO can delay age-dependent changes to neuromuscular junction morphology, suggesting FOXO is responsible for maintaining neuromuscular junction integrity during aging [34]. Therefore, increasing evidence indicates that both drug- or diet-activated FOXO and overexpression of FOXO slow down aging or muscle aging and reduce ARD of the skeletal muscle and heart.

In this study, we found the muscle FOXO gene played a key role in regulating the aging of the skeletal muscle, and some of these findings were similar to those of other researchers. Besides, the muscle FOXO gene also played a key role in HSI-induced ARD of the skeletal muscle and heart, and it also played a key role in E against ARD of the skeletal muscle and heart. More importantly, we also found that the muscle FOXO gene played a key role in E against HSI-induced ARD of the skeletal muscle and heart. Their mechanism is related to the regulation of the FOXO activity on muscle FOXO/PGC-1α/DSH and FOXO/SOD pathways.

In flies, PGC-1α plays an important role in regulating the function of the skeletal muscle and cardiac muscle since PGC-1α is required for the expression of multiple genes encoding mitochondrial proteins. For instance, it has been reported that the reduction of PGC-1α expression levels in flies acutely compromises negative geotaxis ability and reduces exercise-induced improvement in both negative geotaxis and time to exhaustion. Conversely, muscle/heart-specific PGC-1α overexpression improves negative geotaxis and cardiac performance in unexercised flies [35]. Besides, the PGC-1α is a key regulator of mitochondrial function and muscle fiber specification in the skeletal muscle. dRNF34 plays an important role in regulating mitochondrial biogenesis in the muscle and lipid metabolism through PGC-1α [36] as well. Overexpression of the *Drosophila* homolog PGC-1α is sufficient to increase mitochondrial activity. PGC-1α mutants show mitochondrial respiration defects when complex II of the electron transport chain is stimulated. PGC-1α mediates mitochondrial activity, cell growth, and transcription of target genes in response to insulin signalling [37]. Moreover, tissue-specific overexpression of PGC-1α in stem and progenitor cells within the digestive tract extends lifespan. Long-lived flies overexpressing PGC-1α display a delay in the onset of aging-related changes in the intestine, leading to improved tissue homeostasis in old flies [38]. Finally, 28% (995) of the nutrient-responsive genes were regulated by activated FOXO, including PGC-1α. Mitochondria biogenesis is linked to insulin signaling via dFOXO-mediated repression of a PGC-1α homolog [39]. Aging decreases mitochondrial content and reduces ATP levels [40]. Increased levels of dysfunctional mitochondria within the skeletal muscle are correlated with numerous age-related physiopathological conditions [40].

FOXO is involved in the regulation of oxidation and antioxidant balance, which is closely related to aging in flies. For example, the activation of c-Jun N-terminal kinase (JNK) signaling in neurons increases stress resistance and extends life span in part through FOXO-mediated transcription in Drosophila. JNK/FOXO signaling extends life span via the amelioration of oxidative damage and the mitochondrial dysfunction in neurons [34, 41]. Besides, overexpression of the cardiac dSir2 gene reduces heart oxidative stress and delays heart aging via upregulating cardiac dSir2/Foxo/SOD pathway [15]. The expressions of anti-aging genes such as SOD2, FOXO, and Thor are systemically increased as a consequence of heart-specific Rpd3 downregulation. Showing higher resistance to the oxidative stress, the heart-specific Rpd3 downregulation concurrently exhibited improves cardiac functions, decreases heart failure, and accelerates heart recovery [42].

In wild-type flies, exercise training delays muscle aging while HSI promotes muscle aging. For instance, about 2.0 and 2.5 h of exercise per day displayed a reduced incidence of fibrillation events. Only doing physical exercise continuously for a 2.5-h period increased fractional shortening and total sleep time in Drosophila [43]. Both normal expression and overexpression of the CG9940 resulted in positive influences on the adaptation of cardiac functions, mobility, and lifespan to exercise in aging Drosophila such as exercise slowed the age-related decline of cardiac function, mobility, and extent of lifespan in these flies [44]. Exercise training in young Drosophila reduces age-related decline in mobility and cardiac performance [45]. Exercise training resisted HSI-induced heart presenility via blocking CG2196(salt)/TOR/oxidative stress and activating dFOXO/PGC-1α [20]. Although the PGC-1α is a key regulator of exercise training in flies [35], the FOXO function in the skeletal muscle and heart remains unclear. In this research, our results proved that muscle FOXO played a key role in exercise training resistance to HSI-induced ARD of the skeletal muscle and heart.

Finally, these results suggested that the muscle FOXO expression played an important role in the resistance of exercise training to HSI-induced lifespan shortening. Overexpression of the muscle FOXO gene prolonged the life of flies, but it did not resist the HSI-induced lifespan shortening. Exercise training combined with overexpression of FOXO improved the longevity in HSI flies. Muscle FOXO RNAi decreased the lifespan of flies, and it further decreased longevity when combined with HSI. Exercise training improved HSI-induced shortening of lifespan in muscle FOXO RNAi flies. The skeletal muscle and heart are very important organs for mammals or fruit flies, and their pathological changes are important factors leading to the death of elderly individuals. Increasing or decreasing the skeletal muscle and heart function can reduce or increase mortality in elderly individuals [42, 46, 47]. Although muscle FOXO overexpression can effectively resist salt-induced premature aging of the heart or skeletal muscle, the effects of high salt on the whole body and other vital organs cannot be resisted by muscle FOXO overexpression. The muscle FOXO overexpression cannot ameliorate the increase in salt-induced mortality [48, 49]. However, exercise training induces a systemic adaptation that resists salt-induced shortening of lifespan, but this exercise adaptation can be blocked by muscle FOXO RNAi.

## Conclusion

**Fig. 9:**
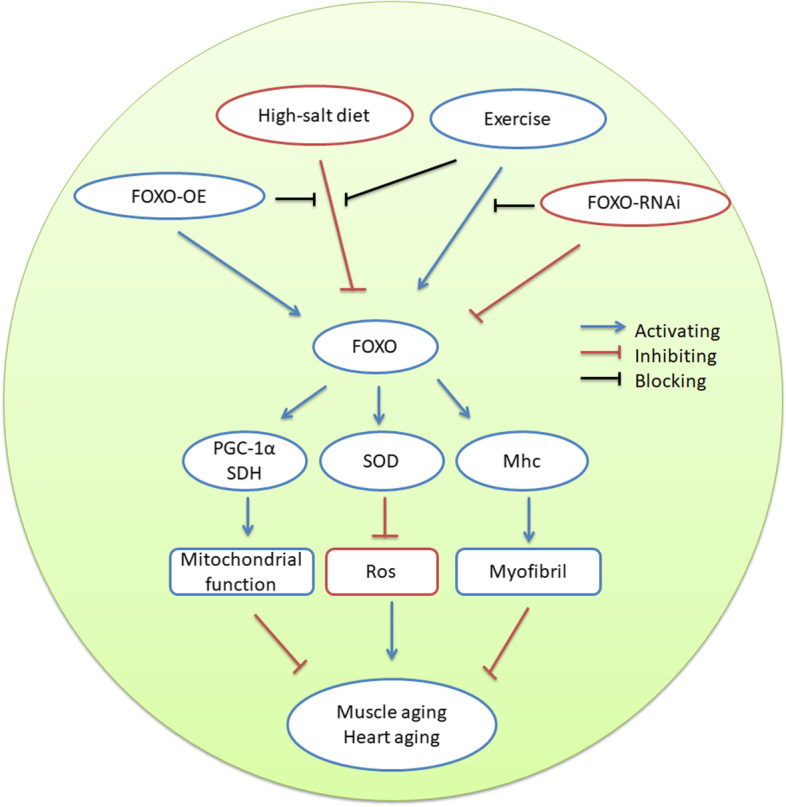
The relationship between HSI, exercise, and muscle FOXO gene on the skeletal muscle and heart. HSI promotes the skeletal muscle and heart aging by inhibiting muscle FOXO-related pathway activity, but FOXO-OE and E can block this physiological process. E can improve the skeletal muscle and heart aging by activating muscle FOXO-related pathway activity, but this physiological process can be blocked by FOXO-RNAi. So, the muscle FOXO gene played a vital role in E against ARD of the skeletal muscle and heart induced by HIS because it determined the activity of muscle FOXO/SOD and FOXO/PGC-1α/SDH pathways in E and HSI flies

Current results confirmed that the muscle FOXO gene played a vital role in E against ARD of the skeletal muscle and heart induced by HIS because it determined the activity of muscle FOXO/SOD and FOXO/PGC-1α/SDH pathways (Fig. 9). The muscle FOXO gene also played an important role in exercise against HSI-induced shortening of lifespan in aging flies.

## Data Availability

All the generated data and the analysis developed in this study are included in this article.

## Abbreviations

FOXO: Forkhead transcription factor
E: Regular exercise
HSI: High-salt intake
FOXO-RNAi: Muscle-specific FOXO-RNAi
FOXO-OE: Muscle-specific FOXO-overexpression
PGC-1α: Peroxisome proliferator-activated receptor-gamma coactivator (PGC)-1alpha
SDH: Succinodehydrogenase
SOD: Superoxide dismutase
ROS: Reactive oxygen species
GSH: Glutathion peroxidase
Mhc: Myosin heavy chain
TTF: Time to fatigue
CI: Climbing index
ARD: Age-related defects
